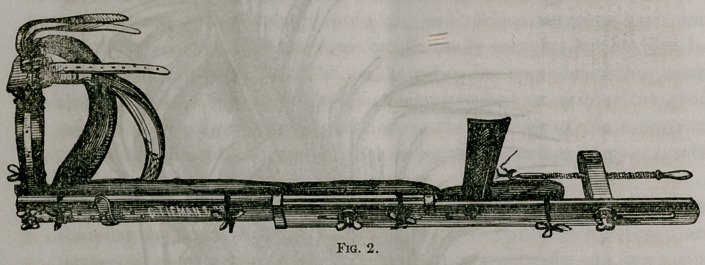# Description of a Modification of the New York Hospital Apparatus for Fracture of the Thigh

**Published:** 1859-06

**Authors:** Frederick D. Lente

**Affiliations:** Surgeon to West Point Foundry, Cold Spring


ART. III.—*Description of a Modification of the New York Hospital Apparatus for the Treatment of Fracture of the Femur: By* FREDERICK D. LENTE, M. D., Surgeon to West Point Foundry, Cold Spring; *With the results of the Treatment of seventy-four Cases of this Fracture.* Furnished by Gurdon Buck, M. D., Senior Surgeon of the Hospital.

An efficient Splint for the treatment of Fracture of the Femur seems still to be a desideratum in the surgical world. Dr. H. Hartshorne, in recently introducing a new contrivance to the College of Physicians and Surgeons of Philadelphia, says—"In examining the excellent report on the treatment of fractures by Dr. Hamilton, in the transactions of the American Medical Association for 1857, we do not find, in the number of contrivances for fractured thigh there described and illustrated, any which meets fully the indications above described." The following are the indications to which he refers.—"That the apparatus should possess fixity and *firmness;* that the extension and counter-extension should be in *the same line;* that their amount and extent should be under *easy* and *exact control*; and, that the *tuberosity of the ischium* is the centre of the most desirable region for counter-extension, as it is best adapted, by structure, and function, for continued pressure." Admitting the propriety of the above conditions, I would suggest two others, which, though not *essential* to the success of the apparatus in an individual case, are absolutely so to its general introduction into surgical practice—viz., that it should be neither *cumbrous* nor *expensive.*

It is my purpose now respectfully to present to the profession an apparatus which seems to approach nearer the fulfilment of these conditions than any other yet described. In order to give due credit to the ingenuity of those surgeons who have, from time to time, been instrumental in building up and modifying this splint, I will, as briefly as possible, trace its history, the changes through which it has passed since its original introduction, more than half a century ago, by Desault, to the present time.

As first used by this surgeon, it is well known that it consisted essentially of an outside splint extending from the crest of the *ilium* to below the sole of the foot. The pressure of the counter-extending band came upon the groin, the ends passing respectively above and below the thigh, and then secured to the upper end of the outer splint. It was Desault's idea to bring the pressure mainly upon the tuberosity of the ischium, but, owing to the faulty attachment of the posterior portion of the perineal band, he did not fully succeed, and most of the stress was borne by the groin and upper part of the thigh, including its great vessels and nerves. Boyer adopted this apparatus, and added the *screw** for making extension in a more uniform and efficient manner; but this failed to obviate the disadvantages of the counter-extension. In order to *fix* more firmly the upper end of the long splint, and to bring the direction of the counter-extending band more nearly in a line with the axis of the limb, and thus take off, to some extent, the pressure upon the muscles and great vessels of the upper part of the thigh, Dr. Physic modified the apparatus in use at the Pennsylvania Hospital by extending the outer splint to near the axilla, the body band then passing across the thorax; he also added a block to this splint below the sole of the foot, in order to bring the direction of the extending force also in a line with the axis of the limb. But the screw was not made use of in this apparatus.

In 1837, Dr. Gurdon Buck, of the New York Hospital, to whom this institution is indebted for a great variety of improvements in its appliances for the treatment of fractures and other injuries, introduced there Physic's modification of Desault, as a substitute for the double-inclined plane, which had, up to that time, as Dr. Buck informs us, been almost exclusively employed in the treatment of fractured thighs. It was not long before this apparatus underwent considerable alterations in the New York Hospital. The *splint-cloth,* always a troublesome necessity, was dispensed with, and its place more advantageously supplied by bands attached to the outside and inside splints respectively. Each band passed from its splint under the limb, around in front, crossing its fellow of the opposite side both behind and in front before being tied, thus maintaining the parallelism of the splints. The cushions also, instead of being loose, were *attached* to the splints. The body belt, which, in the Philad. app. was attached to the long splint, was now disconnected, and furnished, on either side, with a leather pocket opening downwards, which received the end of the splint, and allowed the removal of the latter independently both of the belt and the perineal band, which was attached at each end by a buckle to the lower side of the pocket, before and behind. These modifications were suggested by Dr. Buck. "By this means, the exposure and examination of the limb, as also the reädjustment of the apparatus, could be effected with the least possible disturbance." The screw had already been introduced, and a great improvement also made in the mode of attaching it to the splint, by Mr. Marriner, carpenter to the Hospital; he caused the block, in which the screw plays, to slide in a mortise rep-

* It would appear from some remarks by the editor of Boyer's work on the *bones*, that some imperfect method of extension by the screw had been previously used by Hook and Aitken.

resented at (M.,) Fig. 1, securing it at any required point by a binding screw. The difficulty was now to contrive some safer and more comfortable mode of attaching the extending band to the limb than the old fashion of Desault and Boyer still in use. Extension, when made in this manner, (by a handkerchief, or band, or gaiter about the ankle,) was kept up, in our apparatus, by adjusting pads accurately above the malleoli, and then binding the two splints firmly together; this necessitated a reädjustment daily for three or four weeks. "At length the long sought desideratum in our method was supplied by the employment of bands of adhesive plaster for making *constant traction* upon the limb." * The first suggestion of this important improvement has been ascribed to several different surgeons, but, I believe justly, to Dr. E. Wallace, of Phila., though it was subsequently brought more generally before the notice of the profession, and applied in a more efficient manner by Dr. Josiah Crosby, of New Hampshire. It was through him I first became acquainted with it, and employed it in the New York Hospital in 1849 or '50, while Resident Surgeon there. Finding that the pressure of the plaster on the *malleoli* caused pain and ulceration, I proposed the use of the block now in general use for preserving the parallelism of the bands below the sole of the foot, thus completely relieving the *malleoli* of all pressure. I am not aware that this had been previously used elsewhere, though it is not improbable. With this apparatus, we have certainly obtained, in the New York Hospital, as good results, at least, as have been truthfully† claimed by others, as the statistics kindly furnished me by Dr. Buck, and which will presently be given, abundantly prove. I am not sure that this apparatus, with the alterations and additions now suggested by me, will im-

* The quotations in this article not otherwise credited, are from a letter of Dr. Buck to the writer.

† It is proper to state that it is not meant, by this expression, that surgeons have wilfully misrepresented results. A statement of the following incident, which occurred during the past summer, will explain precisely its bearing: A young American Surgeon, thoroughly drilled in the treatment of fractures, was walking around the wards of a large London Hospital, when the Surgeon in attendance, one of the most eminent in the metropolis, and the author of a deservedly popular work on Surgery, stopped at the bedside of a patient convalescing from fracture of the thigh. The apparatus was removed, the patient laid straight in bed, and the two limbs placed precisely parallel, when it was seen that the internal *malleoli* were exactly on a level. The fact of the non-existence of shortening *thus ascertained,* was dwelt upon with great satisfaction by the Surgeon. As soon as the company had retired from the bed, my friend, the American, took out his graduated tape, measured the limbs carefully, and found just *one inch shortening.* And this is not an isolated occurrence. See Malgaigne on Fractures, and an article by Dr. Hamilton, in Trans. of Am. Med. Assoc., for 1857.

prove upon these results. I have, however, good reason to believe that it will. But it will be worn with less discomfort by the patient, and will have even a neater appearance than its progenitor.

The pressure of the counter-extending band upon the groin has always been the stumbling-block of this apparatus. Desault saw the advantage of making the tuberosity of the ischium the *point d'appui*, but failed, as we have seen, in his attempt to do so; and various surgeons have since contrived as many different plans for effectually carrying out his idea, but without complete success. No one, however, has approached this nearer than the Burges. However, the fact seems to be that *neither the groin nor the tuberosity is fitted to bear alone the pressure of the counter-extension* in cases of considerable shortening, and therefore of great tension in the application of the extending power.

It is therefore my object, in the further modification of the New York Hospital apparatus, *to distribute the pressure on these two points;* and further, in order to render the pressure on the groin safer and more comfortable, and also to remove *all* pressure from the muscles, vessels, nerves, &c., of the thigh in front, I propose to add an *iron brace* (A) Fig. 1, extending, in a curved form, from the upper end of the external splint directly across the body to the median line, and cushioned on its inner surface as represented in the engraving. Sliding on this, and furnished with a binding screw to fix it at any required point, is a plate P., to the lower part of which is attached a buckle for securing the anterior extremity of the perineal band. By this arrangement, I am enabled to make the direction of the counter-extending force of this portion of the band correspond to the axis of the limb, instead of oblique; and, furthermore, it allows me to dispense with all that portion of the outer splint between the crest of the ilium and the axilla; thus reducing it to the original length of Desault, obviating the constriction of the chest by the body-band, and producing a less irksome confinement of the upper part of the body. In lieu of the body-band, there is a pelvic strap extending from the end of the iron brace, to the movable plate to which it is secured by buckles, around the back to the top of the splint, thus binding the apparatus firmly to the pelvis, if found necessary. It should be mentioned that the brace is so attached to the splint, through the ingenuity of Mr. Tiemann, Surgical Instrument maker, of New York, as to allow of its being shifted to either side according as the fracture is on the right or left, or of being removed for packing. He has also made the long splint in two portions sliding on each other so as to shorten or lengthen it according to the size of the patient, and to facilitate its package and transportation. Desault attached the posterior as well as the anterior extremity of the perineal band to the long splint; but it will be found that, by so doing, he does not grasp with it, as he intended, the tuberosity; on the contrary, when extension is applied, it slips under it or above it, and is thus almost totally ineffectual in relieving the groin. To be effective, it should be attached

Fig. 1.*

to the splint at a point considerably lower down; and it is necessary that the medium of attachment should be movable, in order that, when the upper end of the splint is placed opposite the *crista ilii*, it may be shifted, if necessary, a trifle upward or downward, that the band may exactly

* The omission of the letters of reference on the figures was noticed too late to have them supplied. The reader can do so, however, without difficulty.—Editor.

grasp the tuberosity. I therefore provide a button (B) Fig. 1, secured by a thumb-screw, and several holes at different contiguous points in the splint, to which it may be shifted with facility. The posterior end of the perineal band is either passed *under* the outer splint and buttoned, as shown at (B) Fig. 1, or carried between the cushion and splint, *over* the top of the latter to the button, as indicated at (C) Fig. 2. The latter ar-

Fig. 2.

rangement is applicable especially to fat and muscular subjects, particularly females, who have an abundance of fat and other tissues covering the tuberosity, which might allow the band to slip by the bone, unless attached in this manner.* I propose also to attach both the extending and counter-extending bands to the apparatus through the medium of *elastics.* Upon suggesting this to Mr. Tiemann, I found that some one had anticipated me with regard to the *extending* band ; and Mr. T. has arranged a strong spiral spring in the ferule of the screw, which supplies the place of the elastic at that point. It is absolutely necessary that the elastics attached to the perineal band, which may be of india rubber, should be *very short,* an inch or so, and very *strong ;* otherwise, they will *give* too much to the extending force, and had better be dispensed with entirely. These elastics are intended to fulfil two indications ; first, to render the pressure more tolerable to the patient, as elastics always do ; secondly, to *keep up* an equable and uninterrupted traction on the muscles of the thigh, thus tending still further to diminish the shortening, and to counteract the effect of any stretching or yielding in any part of the apparatus. In order to render the pressure of the perineal band still less unpleasant, and less likely to cause excoriation of the groin, it might be of service to apply several coatings of a mixture of Collodion 25 parts, Castor Oil 1 part, which has been found to form, in other parts of the body, and might form here a smooth and enduring cuticle.

* In Dr. H. Hartshorne's recent arrangement, the band apparently grasps the tuberosity well, but has the disadvantage of constricting the muscles of the thigh to a considerable extent; and, what is still more objectionable, a tendency to lift the upper fragment forwards.

My remaining modification of the splint is a *foot-piece*, (D) Fig. 1, attached by a slide and thumb-screw to the mortise in the external splint, and capable of removal at pleasure. This is intended to obviate two inconveniencies of the old arrangement; first, to prevent a tendency to *eversion* of the foot, which almost always exists, sometimes to a great extent; and, secondly, by projecting a little beyond the toes, to take off the pressure of the bed-clothes, which tends still further to evert the foot, and is, besides, exceedingly uncomfortable to the patient. In Fig. 2 this arrangement is dispensed with, and its place supplied by a foot-piece, (E) which also obviates the necessity for the block for preserving the parallelism of the adhesive bands, since these bands pass from the leg, on either side, around this piece, binding it firmly to the sole of the foot. The cords connecting it with the screw are so arranged as to draw uniformly on this, so as not to tilt it against the "ball" of the foot. By resting below the heel on the mattress, it serves to support the weight of the clothes, and also prevents eversion of the foot. This contrivance is an imitation of Boyer's, and may, by some surgeons, be preferred; although it is, in my opinion, not so efficient as the foot-piece (D) Fig. 1. (F.) is a wedge-shaped cushion, very useful in maintaining the whole apparatus in a level position, and taking off the pressure from the heel and *tendo Achillis*. An inside splint, extending from the perineum to the inner *malleolus*, and a guttered splint for the upper and lower surfaces of the thigh respectively, with suitable cushions for the splints, complete this apparatus.

### MODE OF APPLYING THE SPLINT.

It is of the utmost importance to the comfort of the patient, and to the full success of the treatment, that *all* the details of its proper application should be thoroughly comprehended and observed. Therefore, at the risk of extending this article to an unreasonable length, and for the benefit of those not practised in application of complicated apparatus, I venture to describe fully the different steps in the adjustment of this to a recent fracture. Having laid the patient on a firm, unyielding mattress, covered, if desirable, with one or two folded blankets, and measured the respective lengths of the sound and the unsound limb, comparing the results of two or more measurements, shave the leg from the knee to the ankle, and apply a strip of adhesive plaster three inches broad and well warmed, to both sides of the leg from just below the tuberosity of the tibia, allowing the strips to extend some inches below the sole of the foot. These free ends are to be warmed, and smoothly stuck to a thin block as wide as the ankle joint; through holes on either side of which, a strong cord is to be passed, to be attached to the hook or ring terminating the screw. A roller bandage is now to be applied from the foot to the groin, taking care to make considerable pressure with it over the adhesive strips. It may be advisable to dispense with the roller to the thigh, in certain cases. The long splint, having been adjusted to the length of the limb, and its cushion secured to it, is to be placed along the outside of the limb, its top just reaching the crest of the ilium; then, having passed the pelvic band under the body, the thigh must be slightly raised by an assistant, and a guttered splint, furnished with a thin cushion, and corresponding in length with that of the thigh, is to be passed under the latter, extending from the tuberosity of the ischium to the popliteal space. At the same time, the perineal band, previously buckled to the iron brace, is to be passed under the thigh just below the tuberosity, and buttoned to the outer splint at a point nearly opposite this, but a little higher up. The extending band is now to be secured to the screw, while an assistant is making moderate traction on the foot, and simultaneously pushing up the long splint; the object of this being to tighten everything before commencing extension with the screw, every turn of which will then give an increase of tension. If, after this, the splint is found to have been forced up a little higher than the *crista ilii,* the perineal band must be buckled, at either end, a little tighter, in order to rectify this. The inside splint may now be applied, and the concavity between its cushion and the leg, a little more than filled up with folds of old blanket, or some such elastic material; so that, when the splints come to be bound firmly together, the inner condyle of the femur may not be painfully pressed upon; a circumstance which is not apt to be attended to, and which frequently causes great discomfort to the patient. A guttered splint, similar to that on the posterior surface of the thigh, must now be placed on the anterior, and then all the splints, with the enclosed limb, are to be encircled by four strips of strong muslin or linen, about an inch and a half wide, and long enough to pass twice around the splints and tie in a bow-knot; two of these for the thigh, and two for the leg. The manner of applying these bands is of considerable importance, as it is thus that we are enabled to dispense with the splint-cloth, and to keep the splints from tilting forward without it. One end is to be passed under the limb, then around it in front, under again, and around in front, to be there tied to the other end; in doing this it is seldom necessary to raise the limb sufficiently to disturb the patient. Having *loosely* tied these bands, it will be better to look again to the adjustment of the perineal strap to the tuberosity of the ischium, before completing the extension, and to alter the position of the movable button, if necessary. Having now made a degree of extension, by the aid of the screw, corresponding to the amount of shortening, the foot-piece is to be screwed on to the mortise of the splint, so as to correspond to the outer surface of the foot. The *bands* are now to be firmly tightened and tied; and if care has been taken to keep the inner condyle of the femur from undue pressure, these bands may be tightened to any required degree without any inconvenience to the patient. Next, the wedge-shaped cushion is to be placed under the apparatus, and finally the pelvic band is to be tightened as firmly as the patient can comfortably bear. I have given these minute details, because a want of attention to one of them, however trivial it may appear, will often so incommode the patient a few hours after the application of the apparatus, as to necessitate the loosening of some part; while the friends, in their ignorance, will often interfere with just that portion which it is most important to leave undisturbed; and, if the surgeon is at a distance, as often happens in country practice, there is a delay of one or perhaps two days before a reädjustment can take place, and thus suits for malpractice constantly originate. Now this I conceive to be one of the prominent advantages of this apparatus,—that it is eminently suited to the country surgeon, as I have learned from personal experience, even before the present improvements were made. For instance, I have applied it to a boy of fifteen, a very restless age, at a distance of several miles, and found it, at an interval of two or three days, exactly as it was left, the patient, in the meantime, having felt almost no inconvenience from pressure. Again, I have applied it to a large, heavy adult, had him removed from the second story of a house down a narrow stair-case on a board, across the street to another house, through several short turns, and laid on a bed, the apparatus remaining just as it had been previously arranged. A patient under treatment with this apparatus can attend to the calls of nature with very little inconvenience; he has only to raise his hips slightly, with the aid of the leg and arm of the sound side, while the bed-pan is slipped under him, without deranging the apparatus in any degree;* and it is during the performance of this duty that another advantage of my alteration of the perineal band is noticed, since it is thus removed from the immediate vicinity of the anus, where it was always certain to get more or less soiled.

---

* These operations, removing the patient, defecation, &c., could not be performed without great danger of disarranging the splints were they confined together, and their parallelism secured by a board at their lower end, as advised by Hartshorne, Gibson, Flagg, and others, since it is impossible to secure the splints firmly to the limb.

DR. BUCK'S STATISTICS.

After carefully scrutinizing over *one hundred* cases of fracture of the Femur, taken from the Register of the N. Y. Hospital, and eliminating such as involved the cervix, or condyles, or belonged to the class of compound fractures, there remained an aggregate of seventy-four cases, of both sexes, and of all ages from 3 to 63, in which the shaft of the femur alone was fractured. In all these cases, the difference in the length of the fractured limb resulting from the treatment was ascertained by careful measurement with a graduated tape, and the following deductions were drawn from the analysis:

Of the 74 cases of all ages, 19 resulted *without any shortening*, a proportion of about *one-fourth.* The average shortening of the remaining 55 cases, was a fraction less than $\frac{3}{4}$ of an inch.

*Seventeen* cases in the above aggregate were under 12 years of age, of which six resulted without any shortening, a proportion of about *one-third.* The average shortening in the remaining 11 cases, was a fraction less than one-half an inch.

Of the 57 cases over 12 years of age, 13 resulted without any shortening, a proportion of about *one-fourth;* and the average shortening in the remaining 44 cases was a fraction over $\frac{3}{4}$ of an inch.

The above statistics of Dr. Buck, give even a more favorable view of the results of treatment of fractured femur in the New York Hospital, than was claimed by myself in an article on this subject, published in the New York Journal of Medicine, in 1851. I then stated that the *average amount* of shortening was $\frac{3}{4}$ of an inch; but, as I had no statistics of the measurements at that time to guide me, I only gave my own impression, and was therefore careful not to claim too much. These statistics show the average amount of shortening in the whole number, to be about *one-half an inch.* Many of these cases occurred before the introduction of the adhesive plaster extension.

## Figures and Tables

**Fig. 1. f1:**
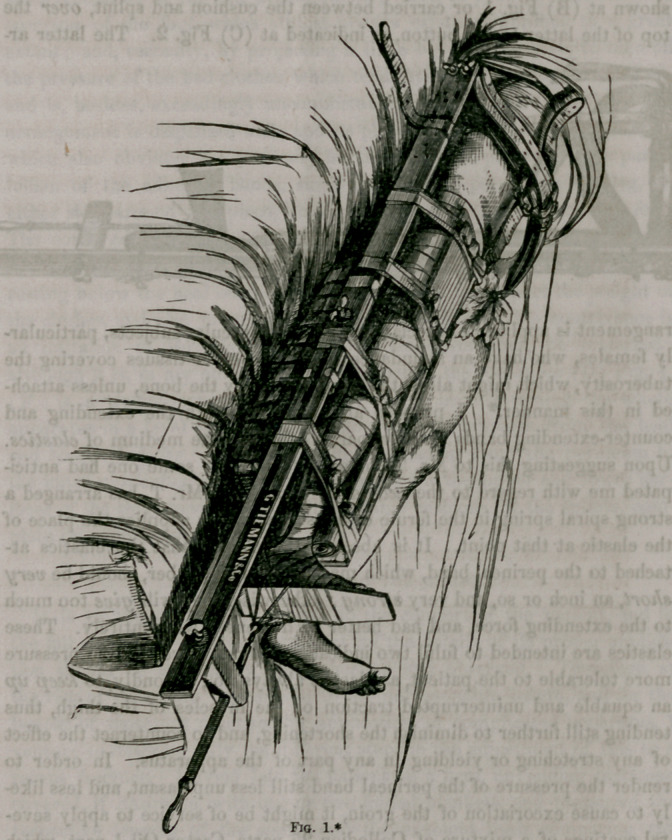


**Fig. 2. f2:**